# Uncommon Fatty Acids and Cardiometabolic Health

**DOI:** 10.3390/nu10101559

**Published:** 2018-10-20

**Authors:** Kelei Li, Andrew J. Sinclair, Feng Zhao, Duo Li

**Affiliations:** 1 Institute of Nutrition and Health, Qingdao University, Qingdao 266021, China; likelei@qdu.edu.cn (K.L.); fzhao@qdu.edu.cn (F.Z.); 2 Faculty of Health, Deakin University, Locked Bag 20000, Geelong, VIC 3220, Australia; andrew.sinclair@deakin.edu.au; 3 Department of Nutrition, Dietetics and Food, Monash University, Notting Hill, VIC 3168, Australia

**Keywords:** furan fatty acids, docosapentaenoic acid, conjugated fatty acids, cardiovascular disease, metabolic disease, blood lipids, inflammation, antioxidant

## Abstract

Cardiovascular disease (CVD) is a major cause of mortality. The effects of several unsaturated fatty acids on cardiometabolic health, such as eicosapentaenoic acid (EPA) docosahexaenoic acid (DHA), α linolenic acid (ALA), linoleic acid (LA), and oleic acid (OA) have received much attention in past years. In addition, results from recent studies revealed that several other uncommon fatty acids (fatty acids present at a low content or else not contained in usual foods), such as furan fatty acids, *n*-3 docosapentaenoic acid (DPA), and conjugated fatty acids, also have favorable effects on cardiometabolic health. In the present report, we searched the literature in PubMed, Embase, and the Cochrane Library to review the research progress on anti-CVD effect of these uncommon fatty acids. DPA has a favorable effect on cardiometabolic health in a different way to other long-chain *n*-3 polyunsaturated fatty acids (LC *n*-3 PUFAs), such as EPA and DHA. Furan fatty acids and conjugated linolenic acid (CLNA) may be potential bioactive fatty acids beneficial for cardiometabolic health, but evidence from intervention studies in humans is still limited, and well-designed clinical trials are required. The favorable effects of conjugated linoleic acid (CLA) on cardiometabolic health observed in animal or in vitro cannot be replicated in humans. However, most intervention studies in humans concerning CLA have only evaluated its effect on cardiometabolic risk factors but not its direct effect on risk of CVD, and randomized controlled trials (RCTs) will be required to clarify this point. However, several difficulties and limitations exist for conducting RCTs to evaluate the effect of these fatty acids on cardiometabolic health, especially the high costs for purifying the fatty acids from natural sources. This review provides a basis for better nutritional prevention and therapy of CVD.

## 1. Introduction

Cardiovascular disease (CVD) is a major cause of mortality [1]. In addition to genetic factors, dyslipidemia [2], poor glycemic control [3], oxidative stress [4], inflammation [5], obesity [6], hyperhomocysteinemia [7], smoking [8], lack of exercise [8], and dietary factors [8] are all related to the development of CVD.

Fatty acids are important structural components of biological membranes and an energy source for living organism. In addition, they also play an important role in regulating many physiological processes, such as inflammation, glycemic control, lipid metabolism and oxidative stress. All these physiological processes are closely related to the development of CVD and metabolic disorders. The beneficial effect of several unsaturated fatty acids, such as eicosapentaenoic acid (EPA) docosahexaenoic acid (DHA), α linolenic acid (ALA), linoleic acid (LA), and oleic acid (OA), on cardiometabolic health has been given much attention [9,10,11,12,13,14,15,16,17,18,19]. However, the effect of several other uncommon fatty acids (fatty acids present at a low content or else not contained in usual foods) on cardiometabolic health, such as furan fatty acids, *n*-3 docosapentaenoic acid (DPA), and conjugated fatty acids, is also worthy of attention. In the present review, we searched PubMed, Embase, and the Cochrane Library for the terms (docosapentaenoic acid OR *n*-3 polyunsaturated fatty acids OR DPA OR furan fatty acids OR CMPF OR conjugated linolenic acid OR conjugated linoleic acid OR conjugated fatty acid OR CLNA OR CLA) in combination with (cardiometabolic health OR cardiovascular disease OR CVD OR blood lipids OR diabetes mellitus OR glucose metabolism OR oxidative stress OR inflammation) up to July 2018, to review the research progress on the effect of these uncommon fatty acids on cardiometabolic health. Studies in humans, animals or in vitro evaluating the relationship between uncommon fatty acids (furan fatty acids, conjugated fatty acids or DPA) and cardiometabolic health were included in the present review. The review should provide a basis for better nutritional prevention and therapy of CVD and metabolic disorders.

## 2. Furan Fatty Acids and Cardiometabolic Health

### 2.1. Origin of Furan Fatty Acids

**Figure 1:**
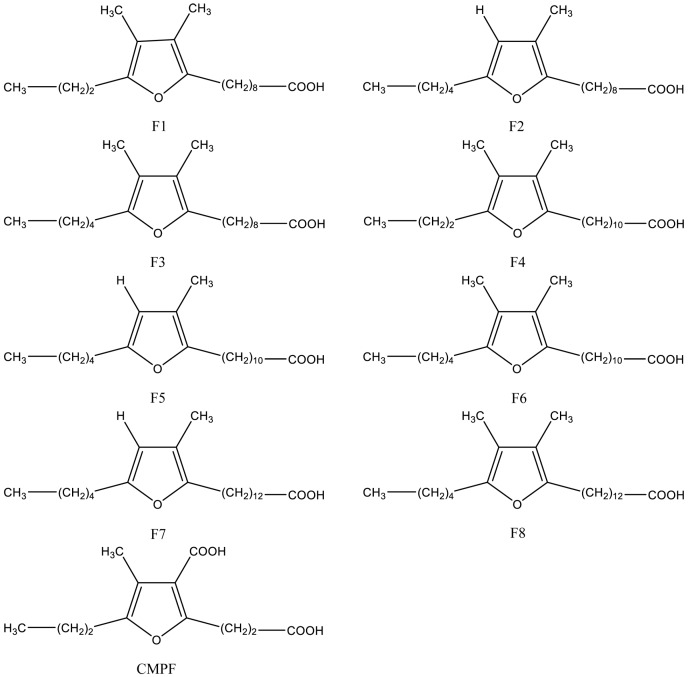
Structure of most abundant furan fatty acids (F1–F8) [35] and their bioactive metabolite 3-carboxy-4-methyl-5-propyl-2-furanpropionic acid (CMPF).

Naturally occurring furan fatty acids are a family of fatty acids consisting of a furan ring with a fatty acid chain on the α1-position, a short straight alkyl group on the α2-position, a methyl group on the β1-position, and either a methyl group or a hydrogen atom on the β2-position [20]. Structures of the most abundant furan fatty acids are shown in Figure 1. Furan fatty acids have been found in different biological species, in low concentrations, including marine and freshwater fish [21], plants [22], algae [23,24], crustaceans [25,26], mammals [27], human tissues [28], fungi [29], ascidian [30], bacteria [31], bivalves [32], and echinoidea [33]. Furan fatty acids are mainly enriched in cholesterol ester and fraction of triacylglycerol (TAG) in liver of mammals, but occur mainly in phosphatidylcholine (PC) and phosphatidylethanolamine (PE) in plasma [34].

### 2.2. Evidence from Human Studies

In addition to EPA and DHA, furan fatty acids may be another bioactive constituent of marine food and its oil product beneficial for cardiometabolic health despite its low concentration (around 1% in some species of fish [21,28]). Fish oil consumption significantly increased 3-carboxy-4-methyl-5-propyl-2-furanpropionic acid (CMPF), a metabolite of common furan fatty acids, by 3-fold and 5 to 6-fold in serum and urine of humans, respectively [36]. Our recent randomized controlled trial (RCT) in subjects with type 2 diabetes mellitus (T2DM) indicated that fish oil consumption significantly increased the serum CMPF level, and most importantly, the change of CMPF during intervention was negatively correlated with the change of plasma TAG levels [37]. In another randomized crossover study, a multifunctional diet (MFD) formulated according to the Nordic Nutrition Recommendations improved serum total cholesterol (TC), low density lipoprotein cholesterol (LDLC), triacylglycerol (TAG), ratios of LDL to high density lipoprotein (HDL) and apolipoprotein B (ApoB) to ApoA1, glycated hemoglobin (HbA1c), C-reactive protein (CRP) and systolic blood pressure [38]. Metabolomics analysis indicated that this MFD significantly increased plasma level of four furan fatty acids, including CMPF, 3,4-dimethyl-5-pentyl-2-furanpropanoic acid (a furan fatty acids have been identified in crayfish), and furan fatty acids with molecular weights of 226.084 and 252.172 (accurate structure was not confirmed). Furthermore, a strong inverse correlation of CMPF and 3,4-dimethyl-5-pentyl-2-furanpropanoic acid with TC, LDLC, and the LDLC to HDLC ratio was observed [38]. Furan fatty acids may also have an anti-inflammatory effect: The New Zealand green lipped mussel contains a certain amount of furan fatty acids [32], and our previous RCT indicated a lipid extract from mussels effectively improved clinical conditions of patients with rheumatoid arthritis, significantly decreased levels of tumor necrosis factor α (TNF-α), interleukin 1β (IL-1β) and prostaglandin E2 (PGE2), and significantly increased levels of IL-10 [39].

However, until now, no clinical trials have been conducted to evaluate the effect of furan fatty acids with relatively high purity on cardiometabolic health because they are usually difficult to separate in quantity and high purity from other fatty acids. One previous study indicated that starvation could dramatically increase the weight percentage of furan fatty acids in total lipids of cod liver by more than 34% [21]. A method for isolation of furan fatty acids from fish lipids has been reported, and the process included: lipid extraction by chloroform and methanol (2:1), methylation of fatty acids, hydrogenation of common straight-chain unsaturated fatty acid methyl esters, and removal of saturated fatty acid methyl esters by crystallization as urea complexes [35]. In addition to isolation of furan fatty acids from lipids of food, artificial synthesis is another option to obtain furan fatty acids with relatively high degree of purity [40].

### 2.3. Evidence from Animal and In Vitro Studies

One recently published animal study indicated that purified CMPF supplementation improved insulin sensitivity, increased beta-oxidation, reduced lipogenic gene expression, and ameliorated steatosis [41]. This suggested that CMPF has a favorable metabolic effect. However, a contradictory result concerning the effect of CMPF on glucose metabolism was observed by another animal study, including glucose intolerance, impaired glucose-stimulated insulin secretion, and decreased glucose utilization [42]. Sand et al. extracted furan fatty acids from northern pike (*Esox lucius*) testes, and supplementation of furan fatty acids to rats led to the appearance of CMPF in urine, indicating that CMPF was a metabolite of common furan fatty acids [43]. Wakimoto et al. extracted furan fatty acids (predominantly F4 and F6) from mussels and compared their anti-inflammatory effect with EPA, commercially available F6, and the anti-inflammatory drug Naproxen in a rat model of adjuvant-induced arthritis, and found that furan fatty acids extracted from mussels had comparable anti-inflammatory effect with Naproxen and commercially available F6; the anti-inflammatory effect of furan fatty acids was more potent than that of EPA [32]. The anti-inflammatory effect of a lipid extract from mussel was also observed in our previous animal study which found that a lipid extract from mussel had an equivalent protective effect to fish oil on intestinal integrity after lipopolysaccharide (LPS) challenge in mice, by increasing the expression of anti-inflammatory cytokine IL-10, decreasing the expression of pro-inflammatory cytokines TNF-α and IL-1β and downregulating toll-like receptor 4 (TLR-4) signal pathway [39]. These results indicated that furan fatty acids, long neglected, might be one bioactive component responsible for the anti-inflammatory effect of lipid products of marine food. Okada et al. synthesized four kinds of furan fatty acids, F2, F3, F6, and 9,12-epoxyoctadeca-9,11-dienoic acid (NMF), and found that tetra-alkyl substituted furan fatty acids (F3 and F6) had the best antioxidant activity in vitro by decomposing hydroperoxides and scavenging peroxyl radicals; the antioxidant activity of tri-alkyl substituted compound (F2) was about 50% as effective as the tetra-alkyl substituted one, while di-alkyl substituted one (NMF) revealed no significant activity [44]. Another in vitro study by Okada et al. compared the hydroxyl radical scavenging activity of artificially synthesized furan fatty acids (F2, F3, and NMF) with other common hydroxyl radical scavengers, and found that the hydroxyl radical scavenging activity of furan fatty acids was better than mannitol and ethanol, and was comparable with that of histidine and dimethyl sulfoxide (DMSO) [45]. Lipid oxidation is an important aspect in the pathogenesis for CVD [46]. Therefore, furan fatty acids, because of their antioxidant activity, may also play a role in protecting against aspects of CVD.

### 2.4. Brief Summary

Evidence from previous studies implied a favorable effect of furan fatty acids on cardiometabolic health. Clinical trials are required to confirm the direct effect of furan fatty acids on cardiometabolic health by using purified furan fatty acids as treatment.

## 3. DPA and Cardiometabolic Health

### 3.1. Origin of DPA

**Figure 2:**
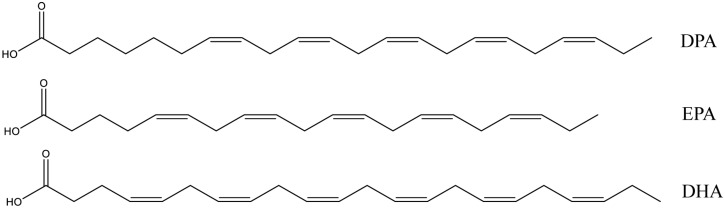
Structure of *n*-3 docosapentaenoic acid (DPA), eicosapentaenoic acid (EPA), and docosahexaenoic acid (DHA).

DPA is a long chain *n*-3 polyunsaturated fatty acid (LC *n*-3 PUFA) widely existing in marine foods and fish oils, together with EPA and DHA. The structures of DPA, EPA, and DHA are shown in Figure 2. The content of DPA in most fish is typically much less than that of EPA and DHA, and in salmon flesh, fish oil, and seal oil the levels are about 0.3%, 2–5%, and 4–5%, respectively [47]. One previous study dramatically increased DPA content in a marine fish, nibe croaker, from 1.8% to 4.1% by addition of an elongase gene to the fish [48].

### 3.2. Evidence from Human Studies

Most previous studies evaluating the effect of LC *n*-3 PUFA on cardiometabolic health did not discriminate between the effect of EPA, DHA, and DPA. Results from recent studies indicated that DPA could also influence cardiometabolic health. Our previous meta-analysis based on 10 prospective cohort studies in 20,460 individuals indicated that the risk of stroke was negatively associated with circulating level of DPA (relative risk (RR) 0.74, 95% confidence interval (CI) 0.60 to 0.92) and DHA (RR 0.78, 95% CI 0.65 to 0.94), but not EPA (RR 0.95, 95% CI 0.82 to 1.12) [49]. Dose–response analysis indicated that 1% increment of DPA and DHA proportions in circulating blood was associated with 25% (RR 0.75, 95% CI 0.64 to 0.87) and 11% (RR 0.89, 95% CI 0.83 to 0.95) reduced risk of stroke, respectively [49]. One meta-analysis based on prospective cohort studies indicated that coronary risk was negatively associated with circulating levels of DPA (RR 0.64, 95% CI 0.47 to 0.87, 7155 subjects in four studies) [50]. Evidence from observational studies indicated that the circulating DPA level was also negatively associated with the risk of cardiovascular mortality (hazard ratio (HR) 0.68, 95% CI 0.52 to 0.89) [51], sudden cardiac death (odds ratio (OR) 0.70, 95% CI 0.51 to 0.97) [52], heart failure (OR 0.81, 95% CI 0.68–0.95) and peripheral arterial disease (OR 0.61, 95% CI 0.45 to 0.82) [53] as well as carotid intimal–medial thickness (β = −0.0002 ± 0.0007 (standard error), *p* = 0.037) [54].

Our previous double blind crossover study found that pure DPA supplementation could significantly reduce postprandial plasma chylomicronemia compared with pure EPA or olive oil supplementation in healthy subjects [55]. Another crossover study found that compared with EPA alone, EPA + DPA supplementation could significantly lower TAG, TC, non-high-density lipoprotein cholesterol, very low-density lipoprotein cholesterol (VLDLC), ApoC3, and proprotein convertase subtilisin kexin type 9 (PCSK9) in subjects with severe hypertriglyceridemia [56]. This trial also reported that EPA + DPA supplementation rather than EPA alone could significantly reduce nile red staining of lipids in monocytes, and that both EPA + DPA and EPA alone significantly reduce monocytes surface markers CD11c, CD36, and CCR5, indicating that DPA might protect against atherosclerosis by inhibiting foamy monocyte formation and modulating monocyte phenotype [57]. In addition, DPA can modulate lipid mediator profile in a different way compared with EPA: in a crossover study, DPA supplementation increased 19,20-dihydroxy-DPA (19,20-DiHDoPE), 7,17-DiHDoPE, and 15-keto-prostaglandin E2, while EPA increased monohydroxy-eicosapentaenoic acids (HEPEs), and there was no overlap in PUFA metabolites formed after DPA and EPA supplementation [58]. One RCT found that EPA + DPA supplementation dose-dependently increased erythrocyte membrane DPA content, and DPA level was inversely associated with serum CRP level. A cohort study in 2547 children with elevated risk of type 1 diabetes indicated that erythrocyte membrane DPA content but not EPA or DHA was negatively associated with the risk of islet autoimmunity [59]. Our cross-sectional study in healthy subjects indicated that after adjusting for potential confounding factors, platelet phospholipid DPA content but not EPA was inversely correlated with mean platelet volume, an independent risk factor for acute myocardial infarction [60].

### 3.3. Evidence from Animal and In Vitro Studies

In a rat model of rheumatoid arthritis, DPA showed a comparable reducing effect with EPA on the progression and severity of arthritic disease as well as pro-inflammatory cytokines, such as IL-17A, IL-1β, IL-6, and TNFα [61]. In addition, DPA and EPA both significantly down-regulated the activation of mitogen-activated protein kinases (p38 MAPK) and nuclear factor-kappa B (NF-κB) pathways and decreased the expression of cyclooxygenase-2 (COX-2), matrix metalloproteinase-2 (MMP-2) and MMP-9 [61]. This can help explain the mechanism for their anti-inflammatory effect. Moreover, pro-resolving mediators derived from DPA can also contribute to its anti-inflammatory effect, but the types of pro-resolving mediators derived from DPA were different from those derived from other LC *n*-3 PUFA, such as EPA and DHA [62]. In mice fed a high-fat diet, purified DPA, EPA, or DHA supplementation improved serum TC, glucose, and liver cholesterol content; only DPA improved insulin resistance rather than EPA or DHA. DPA and DHA but not EPA prevented decreased serum adiponectin and increased serum alanine aminotransferase (ALT) [63]. Either high-fat diet or high-fructose diet significantly decreased plasma DPA and DHA level in rats, and both LC *n*-3 PUFAs were positively associated with insulin sensitivity [64]. Our previous study in rat liver cells indicated that DPA could down-regulate a series of genes involved in fat synthesis, including sterol-regulatory element-binding protein-1c (*SREBP-1c*), 3-hydroxy-3-methyl-glutaryl-coenzyme A reductase (*HMG-CoA reductase*), acetyl coenzyme A carboxylase (*ACC-1*), and fatty acid synthase (*FASn*) [65]. This result can help explain the mechanism for the protecting effect of DPA against hypertriglyceridemia. Reduced platelet aggregation by DPA was also observed in our previous in vitro study [66].

### 3.4. Brief Summary

DPA has a favorable effect on cardiometabolic health in a different way to the other LC *n*-3 PUFAs, such as EPA and DHA. However, few intervention studies in humans have been conducted to evaluate the direct effect of DPA on risk of CVD. Well-designed RCTs should be conducted by using purified DPA as treatment to clarify this point.

## 4. Conjugated Fatty Acids and Cardiometabolic Health

### 4.1. Origin of Conjugated Fatty Acids

**Figure 3:**
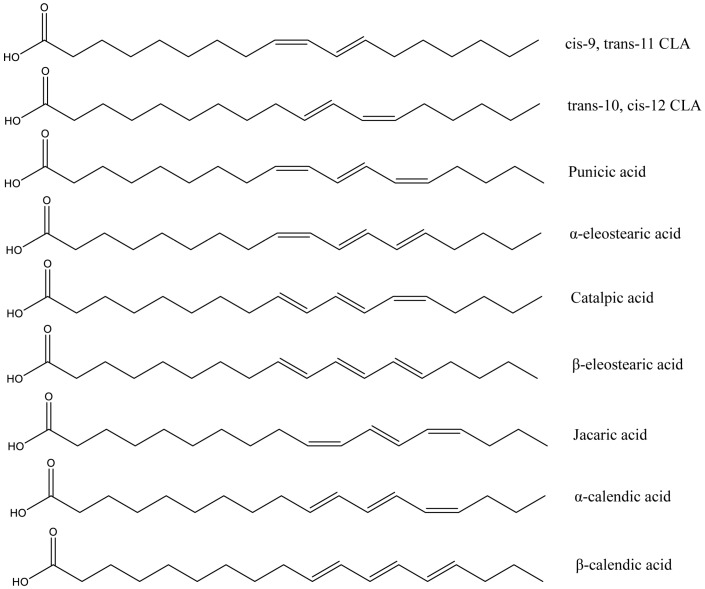
Structure of most abundant conjugated linoleic acids (CLAs) and conjugated linolenic acids (CLNA).

Conjugated fatty acids is the general term for positional and geometric isomers of polyunsaturated fatty acids with conjugated double bonds [67]. The most common conjugated fatty acids are conjugated linoleic acids (CLAs), such as *cis*-9,*trans*-11 CLA and *trans*-10,*cis*-12 CLA, and conjugated linolenic acids (CLNAs), such as α-eleostearic acid, punicic acid, and jacaric acid. *cis*-9,*trans*-11 CLA is the main naturally occurring isomer of CLA, and it was first found as an intermediate for the conversion from PUFA to saturated stearic acid by rumen bacteria [68]. This explained its presence in food from ruminant-animal origin, such as beef, sheep and goat meat and dairy products [67]. In addition, CLA has also been found in low proportions in some plant oils and seafood [69]. CLNA naturally occurs in plant seeds, such as tung seed, bitter gourd seed, snake gourd seed, pomegranate seed, trichosanthes seed, pot marigold seed, jacaranda seed, and catalpa seed [70,71,72]. Several studies in humans and animals indicated that CLNA could be metabolized into CLA in vivo [73,74]. The structures of the most common conjugated fatty acids are shown in Figure 3.

### 4.2. CLA and Cardiometabolic Health

#### 4.2.1. Evidence from Human Studies

Few significant effects of CLA on CVD and its risk factors have been observed in human studies. Only one case-control study reported a significant negative association between *cis*-9,*trans*-11 CLA in adipose tissue and risk of myocardial infarction [75]. One RCT indicated that 6-month supplementation of *cis*-9,*trans*-11 CLA had no significant influence on blood lipids, glucose, CRP, blood pressure, insulin resistance, body composition and 10-year absolute risk of fatal CVD calculated by the European Systematic Coronary Risk Evaluation (SCORE) formula in overweight and obese subjects [76]. Consistent results concerning CVD risk factors were also observed in another two RCTs in healthy subjects and patients with atherosclerosis [77,78]. In addition, results from RCTs even indicated that *cis*-9,*trans*-11 CLA consumption could increase lipid peroxidation [77,79]. One RCT found that supplementation of another isomer of CLA, *trans*-10,*cis*-12 CLA, significantly increased CRP as well as lipid peroxidation [80]. Only one RCT reported a significant lowering effect of CLA (mixture of *cis*-9,*trans*-11 CLA and *trans*-10,*cis*-12 CLA, 50:50) on CRP in patients with atherosclerosis [81].

#### 4.2.2. Evidence from Animal and in Vitro Studies

Lee et al. first reported that CLA (the isomer of CLA used was not reported) could significantly decrease TC, LDLC, TAG, the ratios of LDLC to HDLC and TC to HDLC and led to less atherosclerosis in rabbits [82]. The protective effect of CLA (*cis*-9,*trans*-11 CLA, *trans*-10,*cis*-12 CLA or their mixture) against atherosclerosis was also observed in other animal studies [83,84,85]. A more recent study in mice indicated that CLA could inhibit platelet deposition, decrease macrophage accumulation and expression of the macrophage scavenger receptor CD36 in the aorta, and increase apoptosis in atherosclerotic lesions, and thus exert a pre-resolving effect on atherosclerosis [86]. Increased expression of peroxisome proliferators-activated receptor α (PPARα) and PPARγ by CLA may be one possible mechanism for its protective effects against atherosclerosis [86]. CLA could also suppress monocyte adhesion in vitro by targetting β2 integrin expression [87]. This may be another mechanism for the anti-atherosclerotic effect of CLA. CLA (mixture of *cis*-9,*trans*-11 CLA and *trans*-10,*cis*-12 CLA) decreased pro-inflammatory cytokines (including IL-1β, IL-6, and TNFα) expression in mammary epithelial cells treated with LPS by inhibiting reactive oxygen species (ROS) production and up-regulating PPARγ expression [88]. A PPARγ-dependent anti-inflammatory effect of CLA was also observed in mice with inflammatory bowel disease [89]. Moreover, reducing pro-inflammatory eicosanoids release by inhibiting COX expression is also involved in the regulatory effect of CLA on inflammatory responses [90,91]. In addition, CLA could inhibit high glucose-induced hypertrophy and contractile dysfunction in adult rat cardiomyocytes by modulating PPARγ activation [92]. However, an unfavorable effect on cardiometabolic health was also observed: high dose of CLA supplementation increased insulin resistance in rats fed either a low fat or a high fat diet [93].

#### 4.2.3. Brief Summary

Although favorable effects of CLA on cardiometabolic health have been observed in animal studies and an observational study in humans, there is little evidence from RCTs, which supports this opinion, and several RCTs even reported unfavorable results concerning lipid peroxidation and inflammation. However, few intervention studies in humans have been conducted to evaluate the direct effect of CLA on risk of CVD. Well-designed RCTs should be conducted to clarify this point. 

### 4.3. CLNA and Cardiometabolic Health

#### 4.3.1. Evidence from Human Studies

Favorable effects of CLNA on lipid metabolism were observed in one RCT: pomegranate seed oil supplementation (rich in punicic acid (*cis*-9,*trans*-11,*cis*-13 CLNA)) significantly lowered serum TAG, the ratios of TAG to HDLC and TC to HDLC, and increased serum HDLC in hyperlipidemic subjects [94]. No studies in humans have evaluated the association between CLNA and CVD or other risk factors.

#### 4.3.2. Evidence from Animal and in Vitro Studies

α-eleostearic acid (*cis*-9,*trans*-11,*trans*-13 CLNA) supplementation significantly lowered blood TAG level in diabetic rats [95]. Pomegranate seed oil supplementation, which is rich in punicic acid (*cis*-9,*trans*-11,*cis*-13 CLNA), significantly lowered TC in broilers [96]. Punicic acid (*cis*-9,*trans*-11,*cis*-13 CLNA) also significantly decreased ApoB100 secretion by human HepG2 cell in vitro [97]. ApoB100 is the essential component of very-low density lipoproteins and positively correlated with the incidence of coronary heart disease and atherosclerosis [97]. Bitter gourd oil supplementation, which is rich in α-eleostearic acid (*cis*-9,*trans*-11,*trans*-13 CLNA), decreased blood level of free cholesterol and increased HDLC in rats [98]. Several animal and in vitro studies reported the antioxidative effect of CLNA. Results from an animal study indicated that α-eleostearic acid (*cis*-9,*trans*-11,*trans*-13 CLNA) supplementation could significantly lower LDL-lipid peroxidation and erythrocyte membrane lipid peroxidation in diabetic rats [95]. One in vitro study also demonstrated that both punicic acid (*cis*-9,*trans*-11,*cis*-13 CLNA) and α-eleostearic acid (*cis*-9,*trans*-11,*trans*-13 CLNA) could more effectively scavenge hydroxyl radical and inhibit lipid peroxidation at lower concentrations than at higher concentrations [99]. Another animal study compared the antioxidant effect of punicic acid (*cis*-9,*trans*-11,*cis*-13 CLNA) and α-eleostearic acid (*cis*-9,*trans*-11,*trans*-13 CLNA) in rats, and found that both could effectively decrease oxidative stress and lipid peroxidation induced by sodium arsenite; the antioxidant effect of α-eleostearic acid was more potent than that of punicic acid [100]. However, contradictory results concerning the antioxidative effect of CLNA was observed in another animal study: supplementation of bitter gourd oil (rich in α-eleostearic acid (*cis*-9,*trans*-11,*trans*-13 CLNA)) significantly increased plasma hydroperoxides level [98]. One animal study evaluated the antioxidative effect of punicic acid at different dose, and found punicic acid could act as both pro-oxidant (dose as 1.2% in total fatty acids) and antioxidant (dose as 0.6% in total fatty acids) [101]. Therefore, differences in dose may help explain the inconsistent results concerning antioxidative effects of CLNA observed in previous studies. In addition, CLNA also has an anti-inflammatory effect. Intake of pomegranate seed oil (rich in punicic acid (*cis*-9,*trans*-11,*cis*-13 CLNA)) effectively inhibited neutrophil-activation and protected against trinitrobenzene sulfonic acid (TNBS)-induced colon inflammation in rats; the mechanism involved inhibiting TNF-α induced priming of NADPH oxidase and myeloperoxidase release [102]. One study reported that α-eleostearic acid (*cis*-9,*trans*-11,*trans*-13 CLNA) ameliorated inflammatory bowel disease in mice by activating PPARγ [103]. Down-regulating pro-inflammatory cytokines expression, COX expression and NF-κB signal pathway are potential mechanisms for the anti-inflammatory effects of CLNA [104,105].

#### 4.3.3. Brief Summary

CLNA may have a favorable effect on cardiometabolic health, but evidence from human studies is still very limited, and well-designed RCTs should be conducted to evaluate the effect of CLNA supplementation on CVD and its risk factors.

## 5. Conclusions and Perspectives

DPA has a favorable effect on cardiometabolic health in a different way to the other LC *n*-3 PUFAs, such as EPA and DHA. Furan fatty acids and CLNA may be potential bioactive components beneficial for cardiometabolic health, but evidence from intervention studies in humans is still limited, and well-designed clinical trials will be required. The favorable effects of CLA on cardiometabolic health observed in animals or in vitro have not been replicated in most studies of humans. However, most intervention studies in humans concerning CLA have only evaluated its effect on cardiometabolic risk factors but not its direct effect on risk of CVD, and RCTs with large sample size are still required to clarify this point. Several difficulties and limitations exist for conducting RCTs to evaluate the effect of these fatty acids on cardiometabolic health, especially the high cost of purifying these fatty acids from natural sources because they occur at low levels in natural oils or foods.
